# Isolation of Human Bone Marrow Non-hematopoietic Cells for Single-cell RNA Sequencing

**DOI:** 10.21769/BioProtoc.5020

**Published:** 2024-06-20

**Authors:** Hongzhe Li, Sandro Bräunig, Stefan Scheding

**Affiliations:** 1 Division of Molecular Hematology and Stem Cell Center, Lund University, Lund, Sweden; 2 Department of Hematology, Skåne University Hospital, Lund, Sweden

**Keywords:** Human bone marrow microenvironment, Non-hematopoietic cells, Bone marrow stromal cells, Flow cytometry, CD271, CD45, CD235a

## Abstract

The intricate composition, heterogeneity, and hierarchical organization of the human bone marrow hematopoietic microenvironment (HME) present challenges for experimentation, which is primarily due to the scarcity of HME-forming cells, notably bone marrow stromal cells (BMSCs). The limited understanding of non-hematopoietic cell phenotypes complicates the unraveling of the HME’s intricacies and necessitates a precise isolation protocol for systematic studies. The protocol presented herein puts special emphasis on the accuracy and high quality of BMSCs obtained for downstream sequencing analysis. Utilizing CD45 and CD235a as negative markers ensures sufficient enrichment of non-hematopoietic cells within the HME. By adding positive selection based on CD271 expression, this protocol allows for selectively isolating the rare and pivotal *bona fide* stromal cell population with high precision. The outlined step-by-step protocol provides a robust tool for isolating and characterizing non-hematopoietic cells, including stromal cells, from human bone marrow preparations. This approach thus contributes valuable information to promote research in a field that is marked by a scarcity of studies and helps to conduct important experimentation that will deepen our understanding of the intricate cellular interactions within the bone marrow niche.

Key features

• Isolation of high-quality human non-hematopoietic bone marrow cells for scRNAseq

• Targeted strategy for enriching low-frequency stromal cells

**Graphical overview**


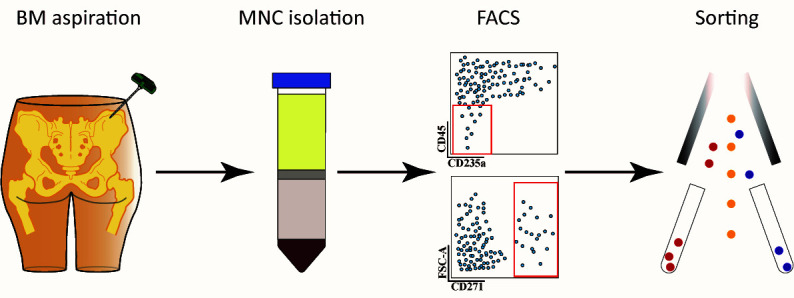


## Background

In human bone marrow, hematopoietic stem cells (HSCs) and their progenies are contained in a specialized microenvironment that regulates HSC maintenance and differentiation. Despite the important role of this hematopoietic environment (HME), its cellular composition, potential heterogeneity, and cellular hierarchy remain poorly defined. This is mainly due to the extremely low frequency of the HME-forming cells, the so-called bone marrow stromal (stem) cells, BMSCs [1,2].

The conventional approach to studying the human bone marrow microenvironment is mainly based on the analysis of different cell types defined by the expression of a limited number of known surface markers, which results in an underestimation of cellular complexity. Novel single-cell–based omics approaches, on the other hand, have the potential to provide detailed insights into complex cellular organization and function. However, whereas bulk preparations of bone marrow cells allow for analysis of the majority of cells, important low-frequency cell populations such as BMSCs will escape detailed analysis. Therefore, we developed a strategy to combine single-cell RNA sequencing of sorted non-hematopoietic BM cells with highly enriched BMSCs to resolve the cellular heterogeneity of the human bone marrow microenvironment at the highest possible resolution based on transcriptomic profiling [3].

Our approach is based on the expression of CD45, CD235a, and CD271. CD45 is a transmembrane protein tyrosine phosphatase encoded by the PTPRC gene (protein tyrosine phosphatase receptor type C). CD45 is considered a pan-hematopoietic marker and is widely used to select all hematopoietic cells and precursors except erythroid cells [4]. CD235a, also known as glycophorin A (GYPA), is a major intrinsic membrane protein of erythrocytes and a distinct marker of erythroblasts [5]. Therefore, we chose to use the combination of CD45 and CD235a to enrich non-hematopoietic human bone marrow microenvironment cells based on their low or absent expression of both CD45 and CD235a. Finally, BMSCs were highly enriched by sorting CD45^low/-^CD235a-/CD271^+^ cells, which is based on data by us and others demonstrating that the CD271 positive BM cell population contains all assayable stromal cells [5–7].

This paper describes a step-by-step protocol to isolate cells from the human bone marrow microenvironment for single-cell RNA sequencing [3] that can certainly be applied to other state-of-the-art omics approaches. Thus, this protocol contributes valuable information that, when combined with future research efforts, will contribute to a deeper understanding of the intricate cellular interactions within the bone marrow niche.

## Materials and reagents

The following materials and equipment are recommended for this protocol, but alternative reagents and equipment from other sources than those recommended herein can be used when shown equivalent.

**Biological materials**

1. Human iliac crest bone marrow aspirates from a healthy donor (*ca.* 50–60 mL), collected in 20 mL syringes prefilled with 1.6 mL of heparin (5,000 IU/mL) (Skåne University Hospital, Lund, Sweden)

**Reagents**

1. Phosphate-buffered saline (PBS) without Ca^2+^ & Mg^2+^ (HyClone, catalog number: SH30256.01)
2. Bovine Serum Albumin (BSA) (Merck, catalog number: A7906)
3. Fetal Bovine Serum (FBS) (Gibco, catalog number: 10270-106)
4. Gammanorm human normal immunoglobulins (Octapharma, catalog number: 096178)
5. Ficoll-Paque Premium (Cytiva, catalog number: 17544203)
6. Mouse anti-human CD45-FITC antibody (BD, clone: 2D1, catalog number: 345808)
7. Mouse anti-human CD235a-PE-Cy5 antibody (BD, clone: GA-R2 (HIR2), catalog number: 561776)
8. Mouse anti-human CD271-APC antibody (Miltenyi, clone: REA844, catalog number: 130-112-602)
9. DAPI stock solution (1 mg/mL) (Sigma, catalog number: D9564)
10. Mouse IgG1-FITC (BD, catalog number: 345815)
11. Mouse IgG2b-PE-Cy5 (BD, catalog number: 555744)
12. Mouse IgG1-APC (BD, catalog number: 345818)
13. BD Pharm Lyse^TM^ Lysing Buffer (BD, catalog number: 555899)
14. Anticoagulant Citrate Dextrose Solution USP (ACD) Solution A (ACDA) (Terumo BCT, catalog number: 77960-010)

**Solutions**

1. Ficoll buffer (see Recipes)
2. Sorting buffer (see Recipes)
3. Blocking buffer (see Recipes)
4. Collection buffer (see Recipes)

**Recipes**

1. **Ficoll buffer**
  PBS with 0.6% ACDA and 2% FBS
2. **Sorting buffer**
  PBS with 1% BSA
3. **Blocking buffer**
  PBS 1:50 Gammanorm, 1% FBS (sterile-filtered)
4. **Collection buffer**
  PBS with 0.04% BSA

**Laboratory supplies**

1. Falcon conical tubes 50 mL (Fisher Scientific, catalog number: 11819650)
2. T-75 culture flask (Merck, catalog number: CLS3290)
3. Filcon 30 μm, sterile, cup-type (BD, catalog number: 340626)

## Equipment

1. Easypet (Eppendorf, catalog number: 4430000018)
2. Centrifuge (Hettich, model: ROTANTA 460R)
3. Cell counter (chemometec, model: NucleoCounter NC-250)
4. Cell sorter (BD, model: Aria II)

## Software and datasets

1. FACSDiva (BD, version 9.0)
2. ChemoMetec NucleoView NC-250 (ChemoMetec, version 1.2.0.0)

## Procedure

- **MNC isolation**
  1. Prepare five 50 mL Falcon tubes with 15 mL of Ficoll-Paque Premium.
  2. Transfer the bone marrow aspirate to a sterile T-75 culture flask.
  3. Add 100 mL of Ficoll buffer to the flask and mix by pipetting up and down.
  4. Carefully layer 30 mL of bone marrow-Ficoll buffer mix over Ficoll-Paque Premium in each 50 mL Falcon tube.
  5. Centrifuge at 300× *g* for 30 min at room temperature without breaks [acceleration rate: 6 (maximum 10); deceleration rate: 0] utilizing a centrifuge equipped with a swinging bucket rotor.
  6. Collect interphases containing mononuclear cells (Figure 1) into five new 50 mL Falcon tubes and fill up with Ficoll buffer to 50 mL.
    **Figure 1.**
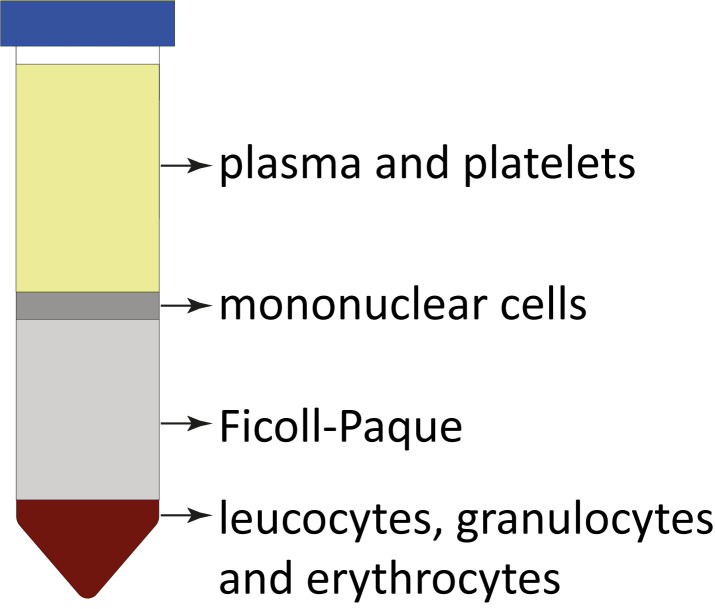
Ficoll-Paque separation demonstration and expected layers after density gradient centrifugation. Each layer represents components with different densities, allowing for the separation and isolation of specific cell populations from the bone marrow aspirates. Cell separation layers starting from the top: plasma; interphase with mononuclear cells; Ficoll-Paque; and bottom fraction with leucocytes, granulocytes, and erythrocytes.
  7. Centrifuge for 15 min at 400× *g* at 4 °C.
  8. Prepare 35 mL of 1× Pharm Lyse by mixing 3.5 mL of BD Pharm Lyse^TM^ Lysing Buffer with 31.5 mL of sterile distilled water.
  9. When centrifugation (step 7) is complete, aspirate the supernatants and re-suspend the pellets by adding 7 mL of 1× Pharm Lyse into each tube and gently pipetting up and down.
  10. Combine the resultant resuspended cell mixtures from the five tubes into one 50 mL Falcon tube.
  11. Gently vortex the sample and keep it for 15 min at room temperature.
  12. Centrifuge at 200× *g* for 15 min at 4 °C.
  13. Carefully aspirate the supernatant.
  14. Resuspend the pellet in 250 μL of Blocking buffer.
  15. Take 5 μL of cells in blocking buffer and mix with 995 μL of Blocking buffer to prepare a 2 to 100 dilution for cell counting.
  16. Count cell numbers using NucleoCounter NC-250 and calculate the original cell number by multiplying with the dilution factor 200.
  17. Adjust the cell concentration to 2 × 10^8^–2 × 10^9^/mL by adding appropriate volume of Blocking buffer.
- **FACS staining**
  1. Incubate cells in Blocking buffer for 20 min at room temperature.
  2. Aliquot the cells and antibodies according to Table 1 to isolate CD45^low/-^CD235a- cells. Aliquot the cells and antibodies according to Table 2 to isolate **CD45^low/-^CD235a-CD271^+^ cells**.
    **Table 1.** FACS staining panel for CD45^low/-^CD235a- cell isolation

Tube ID	Tube name	CD45 FITC (µL)	CD235a PE-Cy5 (µL)	IgG1 FITC (µL)	IgG2b PE-Cy5 (µL)	Cells (µL)	Blocking buffer (µL)
a	Unstained cells	-	-	-	-	5	45
b	Compensation FITC	5	-	-	-	5	40
c	Compensation PE-Cy5	-	5	-	-	5	40
d	*FMO-FITC	-	5	5	-	5	35
e	*FMO-PE-Cy5	5	-	-	5	5	35
f	Sample	25	25	-	-	175	25
    *FMO: fluorescence minus one control
    **Table 2.** FACS staining panel for CD45^low/-^CD235a-CD271^+^ cell isolation

Tube ID	Tube name	CD45 FITC (µL)	CD235a PE-Cy5 (µL)	CD271 APC (µL)	IgG1 FITC (µL)	IgG2b PE-Cy5 (µL)	IgG1 APC (µL)	Cells (µL)	Blocking buffer (µL)
a’	Unstained cells	-	-	-	-	-	-	5	45
b’	Compensation FITC	5	-	-	-	-	-	5	40
c’	Compensation PE-Cy5	-	5	-	-	-	-	5	40
d’	Compensation APC	-	-	5	-	-	-	5	40
e’	FMO-FITC	-	5	5	5	-	-	5	30
f’	FMO-PE-Cy5	5	-	5	-	5	-	5	30
g’	FMO-APC	5	5	-	-	-	5	5	30
h’	Sample	25	25	25	-	-	-	175	-
  3. Incubate the staining tubes for 30 min at 4 °C in the dark.
  4. Wash the stained cells by adding 1 mL of Sorting buffer to the tubes and centrifuge tubes for 5 min at 800× *g* at 4 °C.
  5. Resuspend Tubes a-e (a’–g’ for panel 2) with 500 μL of Sorting buffer and add 2.5 μL of sterile DAPI stock.
  6. Resuspend Tube f (h’ for panel 2) in 3 mL of Sorting buffer and add 15 μL of DAPI stock.
  7. Pass the cells through a 30 μm Filcon or any equivalent strainer.
  8. Proceed quickly with FACS sorting.
- **FACS sorting**
  1. Open the BD FACSDiva Software in the software interface and locate the workspace where you can create plots. Create a plot and choose the parameters to make a Forward Scatter-Area (FSC-A) versus Side Scatter-Area (SSC-A) plot. Adjust the individual FSC and SSC photomultiplier tube settings to visualize the expected cell populations (Figure 2A).
    **Figure 2.**
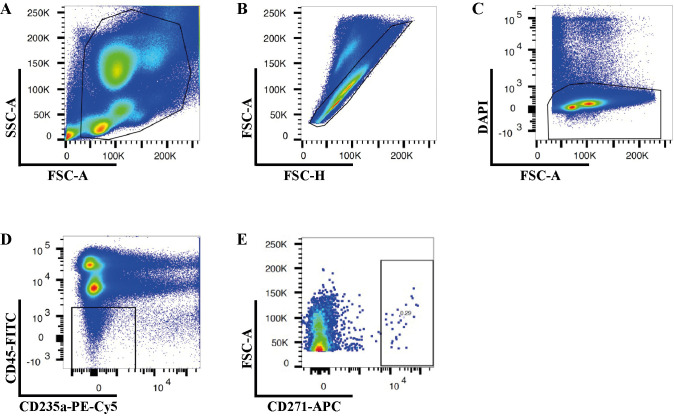
Gating strategies for isolation of human bone marrow CD45^low/-^CD235a- and CD45^low/-^CD235a-CD271^+^ cells. Representative FACS plots illustrate the sequential gating strategy to eliminate cell debris (A), doublets (B), and dead cells (C). Sorting gates for isolation of CD45^low/-^CD235a- (D) and CD45^low/-^CD235a-CD271^+^ (E) cells according to the appropriate FMO controls.
  2. Set up a gate to remove FSC-low populations by drawing a polygonal gate around the regions containing FSC-medium and FSC-high particles, as the FSC-low population consists of cell debris, air bubbles, and laser noise (see Figure 2A).
  3. Create a Forward Scatter-Height (FSC-H) versus FSC-A plot and exclude doublets and multiplets by gating out cells with higher area signal values (FSC-A) (Figure 2B).
  4. Create a FSC-A versus DAPI plot to exclude non-viable cells by gating out the DAPI-high cells (Figure 2C).
  5. Create a FITC versus PE-Cy5 plot to exclude CD45-high and CD235-expressing cells (Figure 2D).
  6. Create a FSC-A versus APC plot to exclude CD271 negative cells (Figure 2E).
  7. Sort 100 events from the CD45^low/-^CD235a- or CD45^low/-^CD235a-CD271^+^ cell fractions into a tube containing 100 µL of ice-cold Collection buffer.
  8. Perform reanalysis with the sorted sample to evaluate the sorting purity (> 85%).
  9. Collect the target cell fraction into a tube containing 700 µL of the ice-cold Collection buffer.
  10. After sorting is complete, count the cell number and re-suspend 20,000 cells with 47 µL of ice-cold Collection buffer.
  11. Proceed immediately to perform single-cell RNA sequencing using Chromium Controller (10× Genomics) and Chromium Single Cell Gene Expression 3’ v3 Reagent Kit.

## Data analysis

To evaluate the effectiveness of cell isolation, various parameters were assessed, including (1) the efficiency of Ficoll-Paque gradient separation, (2) the viability of bone marrow mononuclear cells, (3) the real-time gating strategy utilizing BD FACS Diva, and (4) the efficiency of cell sorting.

1. Ficoll-Paque gradient separation
  After centrifugation, observe the tube to identify different layers. Typically, layers include plasma, a mononuclear cell layer (buffy coat), a Ficoll-Paque layer, and a red blood cell layer. A well-performed Ficoll-Paque gradient separation should result in four distinct layers with the mononuclear cell layer containing a high concentration of nucleated cells. The efficiency of separation can be evaluated by the presence of a clear interface between layers and minimal contamination between different cell populations.
2. Cell viability
  The quality of isolated mononuclear cells can be evaluated using various viability assays. A cell viability of over 90% is recommended, particularly for cell sorting procedures. Cell viability is calculated using the following formula:
  Viability (%) = (1 - Total number of stained cells / Total number of cells) × 100
3. Gating strategy
  An effective real-time gating strategy is paramount for the precise collection of high-quality, viable non-hematopoietic cells for single-cell RNA sequencing. As illustrated in Figure 2, the gating strategy employed ensured the targeted isolation of the desired cell population while meticulously excluding debris (Figure 2A), doublets (Figure 2B), non-viable or damaged cells (Figure 2C), and unwanted hematopoietic cells (Figure 2D). The sequential application of these gates progressively refined the cell population, culminating in the final selection of CD271-positive cells (Figure 2E) for subsequent downstream analyses.
4. Sorting efficiency
  After the initial sorting process, a critical step in ensuring data integrity is the reanalysis of sorted cells. This involves a reassessment of the sorted cell population using the same flow cytometry gating strategy. By performing sorting reanalysis, we could evaluate the sorting purity and efficiency, identify contaminants and refine the gating strategy if necessary. Through meticulous verification and validation, researchers can ensure the consistency and accuracy of sorted cell samples, facilitating trustworthy results in subsequent experiments and analyses. It is advisable to aim for a sorting purity exceeding 85% for optimal suitability in subsequent analyses.

## Validation of protocol

This protocol or parts of it has been used and validated in the following research article:

Li et al. [3]. Identification of phenotypically, functionally, and anatomically distinct stromal niche populations in human bone marrow based on single-cell RNA sequencing. *eLife* (Figure 1, panel A).

## General notes and troubleshooting

**General notes**

1. High-quality BM aspirates should be used fresh and contain sufficient numbers of cells. We routinely collect 50–60 mL of bone marrow from 2–3 aspirations from the same donor. However, lower volumes might also be sufficient. In case BM cells are obtained from biopsies, MNC isolation has to be performed by methods such as “crushing and/or flushing” either with or without the use of enzymes such as collagenase [8,9].
2. CD45 is a transmembrane protein tyrosine phosphatase encoded by the PTPRC gene (protein tyrosine phosphatase, receptor type C). Conventionally, CD45 is considered a pan-hematopoietic marker and is widely used to select for hematopoietic cells. In this protocol, we chose to explore the human bone marrow microenvironment by using bone marrow mononuclear cells that showed low or absent expression of CD45. Our gating strategy aimed to enrich all non-hematopoietic cells but not to exclude potential stromal cells by too rigorous gating. Therefore, several hematopoietic cell types including B cells, NK cells, CD235a- late-stage erythroid progenitors, megakaryocytes, monocytes, dendritic cells, granulocytes, and CD34-expressing putative hematopoietic stem and progenitor cell (HSPC) populations could be identified within this gate. This is consistent with previous murine studies that used comparable gating to enrich non-hematopoietic bone marrow cells, which included multiple CD45- hematopoietic cell populations [10,11]. The hematopoietic cells could be easily identified based on their gene expression profiles.
3. It has been widely known that primary bone marrow stromal cells are difficult to isolate due to the extremely low frequency of this cell type. We therefore recommend including CD271 in the staining panel (Table 2) to further enrich the stromal cells if this is the target population for detailed analysis, as bone marrow stromal stem/progenitor cells are highly and exclusively enriched in CD271-expressing cells [12].
4. Having an effective Ficoll-Paque density gradient separation is one of the crucial steps in obtaining high-quality human bone marrow mononuclear cells for FACS-based cell isolation.
  - Bone marrow samples should be processed for mononuclear cell isolation immediately after aspiration (within 30 min) to achieve the best sample quality with the highest cell viability and cell yield.
  - It is important to use a swing-out rotor (also known as a bucket rotor) instead of a fixed-angle rotor for Ficoll-Paque gradient centrifugation to ensure a better separation of cell layers during centrifugation and a higher cell recovery rate. This is especially crucial when working with limited cell numbers or rare cell populations.
  - In this protocol, we used BD Pharm Lyse^TM^ lysing solution, which is an ammonium chloride (NH_4_Cl)-based lysing reagent, to lyse red blood cells. Incubating cells treated with NH_4_Cl at room temperature for 15 min allows for the complete lysis of red blood cells through osmotic shock. The duration of 15 min for incubating cells has been empirically determined to be sufficient for effective lysis while minimizing damage to other cell types when processing 60 mL human bone marrow samples. The duration of the red blood cell removal step (Procedure A11) can be adjusted depending on the individual sample. Pharm Lyse treatment can be reduced to as short as 2 min if red blood cell contamination is minimal.
5. Although we used CD45-FITC, CD235a-PE-Cy5, and CD271-APC antibodies in this protocol to distinguish CD45/CD235a double-negative cells from the cells that are positive for one or both surface markers and to enrich CD271-expressing cells, any fluorochromes conjugated to these three antibodies could be used to isolate cells from the human bone marrow microenvironment. When selecting antibodies with distinct conjugates, it is advisable to opt for conjugates that have minimal overlap in their emission spectra. As FACS-based cell isolation is highly dependent on the quality of antibody staining, it is recommended to titrate each antibody using bone marrow samples to avoid the antibody saturation effect, optimize signal-to-noise ratio, minimize antibody non-specific binding, enhance consistency and reproducibility, and achieve the best sorting outcome.
6. As the flow cytometric cell sorter plays an essential role in obtaining accurate and reliable results in this protocol, it is extremely important to ensure that the cell sorter is operated under optimal conditions.
  - Machine-specific preparatory steps should be performed according to the manual.
  - The cell sorter should be cleaned properly before the experiment to minimize any potential contamination.
  - Photomultiplier tube (PMT) voltage and detector sensitivity settings need to be adjusted for each fluorochrome.
  - Perform compensation calculation using single-stained compensation controls. Double-confirm the compensation calculation by using FMO controls need to be included.
  - A gating strategy to distinguish positive and negative events based on FMO samples has to be established.
  - The sample flow rate has to be kept within the recommended range to avoid overlapping events (< 3,000 events/second for a 70 µm nozzle, BD Aria II).
  - Ice-cold collection buffer should be used to increase the viability of sorted cells.
7. While our strategy focuses on isolating and characterizing non-hematopoietic cells from the human bone marrow microenvironment using a specific marker panel, it is important to acknowledge a limitation in the underrepresentation of bone-associated cells such as osteoblasts and chondrocytes from the analysis.
8. Lastly, as human samples generally demonstrate high person-to-person diversity, it is recommended to collect as many samples as possible to ensure reproducible results.
